# Development of a flow cytometric panel to assess prognostic biomarkers in fine needle aspirates of canine cutaneous or subcutaneous mast cell tumors

**DOI:** 10.3389/fvets.2023.1279881

**Published:** 2023-11-21

**Authors:** BinXi Wu, Amandine Lejeune, Verena K. Affolter, Giulia Iamone, Fulvio Riondato, Amir Kol

**Affiliations:** ^1^Department of Pathology, Microbiology and Immunology, University of California Davis School of Veterinary Medicine, Davis, CA, United States; ^2^Department of Surgical and Radiological Sciences, University of California Davis School of Veterinary Medicine, Davis, CA, United States; ^3^Department of Veterinary Sciences, School of Agriculture and Veterinary Medicine, University of Turin, Turin, Italy

**Keywords:** flow cytometry, fine needle aspirate, canine cutaneous mast cell tumor, biomarkers, Ki-67, CD117

## Abstract

Mast cell tumor (MCT) is a common skin cancer in dogs that has a wide range of clinical behaviors. The purpose of this study was to develop a novel multicolor flow cytometry (FC) panel that will enable the quantification of candidate prognostic markers (Ki-67 and pKIT) in fine needle aspirate (FNA) samples prior to surgical removal of the tumors. FNA of canine MCTs and the NI-1 cell line were utilized to develop a FC panel that includes a viability dye (FVS620, BD Biosciences; 7-AAD, Invitrogen) and the following primary conjugated antibodies: CD117-PE (ACK45, BD Biosciences), pKIT-A647 (polyclonal bs-3242R, BIOSS) and Ki-67-FITC (20Raj1, eBioscience; MIB-1, DAKO). A total of nine FNA samples of canine MCTs were collected, seven out which produced sufficient cells for FC analysis. The Ki-67 antibody clone 20Raj1 produced a positive signal when applied to blood leukocytes but failed to provide robust labeling of neoplastic mast cells. The Ki-67 antibody clone MIB-1 delivered a superior staining quality in both the NI-1 cells and primary MCT cells. CD117-PE signal was adequate post fixation and permeabilization and in the combination of 7-AAD. pKIT produced non-specific staining and was not suitable for this multicolor FC panel. In conclusion, FNA samples of canine MCTs can often yield adequate cell numbers for FC analysis, and a multicolor FC panel was developed that can detect Ki-67 in canine mast cells. This would permit further studies into the potential use of this panel for canine cutaneous and subcutaneous MCT prognostication purposes.

## Introduction

Canine mast cell tumor (MCT) is a common tumor may occur within the dermis (i.e., cutaneous MCT) or within the subcutaneous fat tissue (i.e., sub-cutaneous MCT) (1, 2), and it accounts for 7–21% of all cutaneous neoplasms (2–4). These tumors have a highly variable biologic behavior ranging from benign to highly metastatic and aggressive (3, 5, 6). An extensive staging diagnostic workup is indicated for cutaneous MCTs if negative prognostic factors are present, such as high tumor grade, but may be unnecessary in low-risk tumors (7, 8). Furthermore, a standardized approach for determining the appropriate extent of lateral surgical margins needed to achieve clear histologic margins remains unavailable, and a tumor of lower grade may not require as extensive a margin to accomplish complete excision (1, 9). While subcutaneous MCTs are considered to have a mostly benign biological behavior (10), a recent study reports a 18% local recurrence rate, a 27% rate of lymph node metastasis and a 13% MCT-related mortality in a cohort of 45 dogs (11). In this context, prognostication assays that can be performed prior to tumor removal are urgently needed.

Ancillary tools have been investigated to help clinicians decide when to be more aggressive in staging and therapy of canine cutaneous MCT. For instance, tumor grade (3, 12) and adjunctive immunohistochemical (IHC) markers such as Ki-67 and phosphorylated c-kit (pKIT) are valuable in predicting the behavior of canine cutaneous MCT. Specifically, the IHC expression level of the nuclear proliferation marker Ki-67 is associated with tumor grade (13) and tumor-related mortality (14). Quantification of pKIT via IHC has been associated with CD117 protein localization, mitotic rate, survival time, tumor grade, and *KIT* mutation at exon 8 and 11 (15). Similarly, Ki-67 may further serve as a useful prognostic indicator in subcutaneous MCTs as well (16). In other words, histologic tumor grading, and biomarker expressions help predict the biological behavior of canine cutaneous and subcutaneous MCTs, providing valuable information on staging and treatment planning, including surgical options and the inclusion of chemotherapy and/or radiotherapy. Unfortunately, histopathology and IHC are most often done following definitive surgery rather than as a preoperative incisional tissue biopsy, thereby limiting their utilities to guide staging and surgical recommendations. Moreover, with the recent commercialization of the intratumoral anti-cancer agent tigilanol tiglate (17), some patients may never get a biopsy or histopathologic analysis of their tumor, further highlighting the need for an accurate, pre-biopsy, prediction of canine MCT biologic behavior.

Fine needle aspirate (FNA) cytology is the primary diagnostic method for canine MCT. Unlike incisional biopsy, FNA cytology is a minimally invasive, affordable, and rapid diagnostic modality with high accuracy in diagnosing MCT (18). It is also a common modality to obtain samples for lymphocyte immunophenotyping via flow cytometry (FC), and has also been used to detect the presence of mast cells in the lymph nodes of dogs with cutaneous MCTs (19). Moreover, FC has been used to quantify Ki-67 expression in canine lymphoma (20). When compared to IHC, additional benefits of using FC to analyze these markers include the potential for more objective, precise, and robust results. For example, a wide range of Ki-67 IHC indices (from 0.01 to 0.135) has been reported as cutoffs for increased risk of mortality and histologic grade in dogs with cutaneous MCTs (13, 21–23), and FC may be better equipped to standardize the cutoff due to a larger number of cells analyzed. Potential limitations of FC include lack of spatial context within the tumor and uneven distribution of biomarker expression within the tumor (24). Finally, FC panels using FNA samples from canine MCTs to quantify Ki-67 and pKIT in canine MCT have not been reported yet. The objectives of this study were to determine if FNA samples of canine cutaneous or subcutaneous MCT can yield sufficient cells for flow cytometric analysis in the clinical setting and to develop a FC panel that includes CD117 (c-kit, a mast cell identity marker) (19), Ki-67, pKIT, and a viability dye.

## Materials and methods

### Cell culture

To optimize the flow cytometric protocol for the quantification of Ki-67 and pKIT in canine neoplastic mast cells, the NI-1 (25) canine mast cell line (abm) was used. This cell line was established from a 3.5-years old mixed breed dog with mast cell leukemia. These cells express both CD117 and pKIT (25). The cells were cultured in an incubator with PriGlow II culture media (abm) or RMPI 1640 (Gibco) with 10% FBS (R&D Systems) and 1% penicillin/streptomycin (Gibco) at 37°C and 5% CO_2_ and passaged every 3–4 days.

### Clinical samples

The study protocol was approved by the University of California, Davis Institutional Animal Care and Use Committee (IACUC protocol #21854). To be included, patients had to be diagnosed with cutaneous or subcutaneous MCT based on FNA cytology. FNA samples from dogs were obtained after an informed consent form was signed by the dog owners. The samples were obtained using a 22ga needle attached to a 3 mL syringe and coated with potassium EDTA. Negative suction was applied after penetrating the tumor followed by redirection of the syringe at least 3 times. Two to four syringes were typically obtained from each tumor. The samples were immediately emptied into a sterile tube containing 1 mL of RMPI 1640 with 10% FBS and 1% penicillin/streptomycin with gentle flushing. Discarded canine whole blood from the hematology diagnostic laboratory at the UC Davis, Veterinary Medical Teaching Hospital was used for individual marker staining optimization. Cell counts and viability on all clinical FNA samples were performed by a Muse Cell Analyzer (Millipore) or a hemocytometer.

### Flow cytometry

Four antibodies and two viability dyes were evaluated individually (see Table 1). Initial optimization of these antibodies was performed using the NI-1 mast cells, with or without the addition of canine peripheral blood leukocytes as an internal negative control. Titration studies were completed to determine the optimal antibody concentration for each antibody. Clinical samples were processed within 2 h of FNA sample collection. Single color controls (AbC total antibody compensation kit, Invitrogen), viability control (1:1 mixture of freshly cultured NI-1 cells and heat-killed NI-1 cells), and fluorescent minus one (FMO) controls were included for every multicolor panel. Flow cytometry data were acquired between 24 and 48 h post staining and fixing for 8/9 of the samples, using a FACScalibur (BD Biosciences). Data for one sample was collected 1 week after staining due to analyzer malfunction. A minimum of 10,000 events (20) in the intact cell gate were acquired. Flow cytometric data were analyzed using a commercially available software (Flowjo).

**Table 1:** List of the antibodies and viability dyes used for flow cytometry analysis of mast cells in this study.

Marker	Clone/reference	Fluorophore	Manufacturer
Viability	N/A	FVS620	BD Horizon
Viability	N/A	7-AAD	Invitrogen
CD117	ACK45 (19)	PE	BD Pharmingen
pKIT	Polyclonal 5401R (15)	A647	BIOSS
Ki-67	20Raj1 (26)	FITC	eBioscience
Ki-67	MIB-1 (13, 20, 22, 23)	FITC	DAKO

### Staining protocol

The samples were incubated, in 100 uL culture media, with anti-CD117 (ACK45-PE) antibody and 7-AAD, at 4°C for 15 min. After incubation, 2 mL of an ammonium chloride-based RBC lysis buffer was used to lyse the RBC and as a wash. This was followed immediately by fixation and permeabilization of the cells (FoxP3 transcription factor fix/perm set, eBioscience) with 30 min of fixation time at 4°C and a single wash using the permeabilization agent. The samples were then incubated at 4°C with anti-Ki-67 (20Raj1-FITC or MIB-1-FITC) antibody and anti-pKIT (Polyclonal 5401R-A647) antibody in 100 uL of permeabilization buffer (with added 2% bovine serum albumin) for 30 min and then followed by a single wash in the permeabilization buffer. 200–300 uL of 1% paraformaldehyde (PFA) in Dulbecco’s phosphate-buffered saline (DPBS) was used to resuspend the samples prior to FC data acquisition. For each run of our final FC panel, 7 tubes were ran as follows: unstained cells, complete panel, FMO-Ki67, FMO-CD117, Viability-only (NI-1 cells), PE single color control (beads), and FITC single color control (beads).

Modifications of the staining protocol were made in an attempt to detect Ki-67 using the 20Raj1 clone. Two different fix/perm reagent kits (FoxP3 transcription factor fix/perm set, eBioscience; True-Nuclear transcription factor fix/perm set, BioLegend) were tested. Modifications of the permeabilization reagent with additional 0.1% Tween, 0.1% Triton, and 0.1% saponin were tested. Different fixation times (30 min vs. 60 min), fixation temperatures (4°C vs. room temperature), antibody incubation times (30 min vs. 60 min), and antibody incubation temperatures (4°C vs. room temperature) were also evaluated.

### Statistics

Commercially available statistical software (GraphPad Prism and Excel) was used for statistical analysis. Descriptive statistics were calculated where appropriate.

## Results

### FNA samples can yield adequate cell number and quality for FC analysis in the clinical setting

Nine FNA samples from dogs with cytologically confirmed MCTs were analyzed for cell yield. Patient demographics, tumor size, location, grade and stage as well as sample collection method and cell yield are detailed in Supplementary Table S1. Six samples were obtained during pre-surgical visits, and three samples were obtained right after surgical excision using the same FNA technique. Tumor size (longest axis) ranged from 0.5 cm to 5 cm. 2–4 syringes were collected from all cases except one where only 1 syringe was collected. Sample cellularity ranged from only 200 to over 16,000,000 total viable cells (200–4,000,000 per syringe) with a median total viable cell yield of 800,000 cells (Supplementary Figure S1). Using 150,000 cells (estimated minimal cells needed for the multicolor panel) as a cutoff, 7/9 (78%) of the samples were adequate for FC analysis. Two out of nine samples (22%) did not yield sufficient cells. Both samples were from tumors that occurred on the limbs/extremities. One of these tumors measured 1.2 cm with only one syringe collected and the other one had measured 0.5 cm. Mild, local adverse events (tumor appeared mildly erythematous or oozing) were noted for two cases and were treated accordingly. Six out of nine samples were used for cell yield analysis and panel development, and 3/9 cases had the full panel performed.

### The anti-CD117-PE antibody (clone ACK45) retains its ability to label canine neoplastic mast cells with subsequent cell fixation and permeabilization

**Figure 1:**
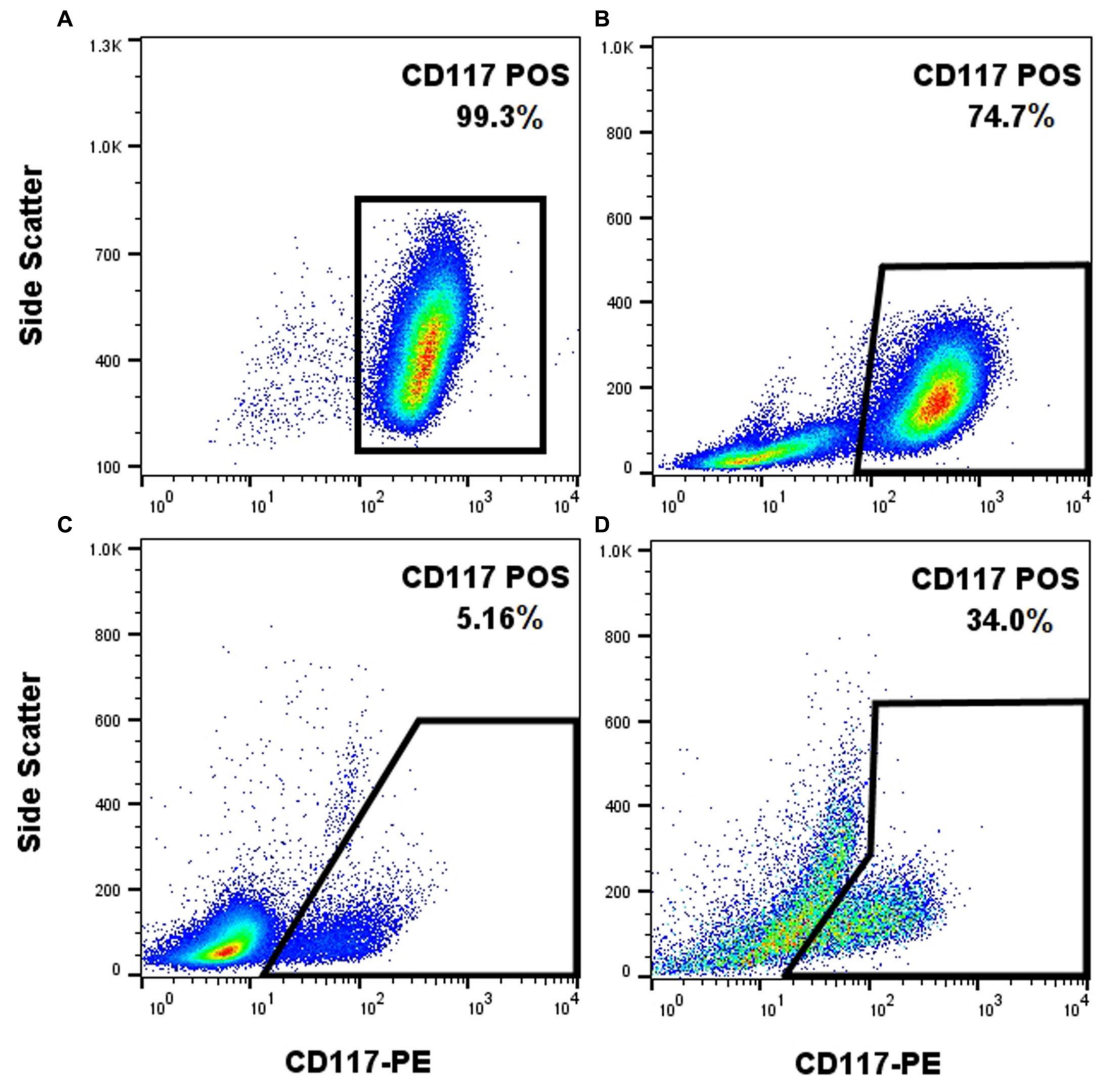
The anti-CD117-PE antibody (clone ACK45, BD Pharmingen) enables effective labeling of canine mast cells with post-labeling fixation and permeabilization. CD117-PE (x-axis) fluorescence plotted against side scatter (y-axis) of different samples containing mast cells. Unfixed NI-1 mast cells showed uniform positivity **(A)**. Fixed and permeabilized NI-1 mast cells spiked with peripheral blood leukocytes showed a discrete population of the mast cells **(B)**. Fixed and permeabilized whole blood from a dog with mastocytemia showed effective separation of mast cells from non-mast cells **(C)**. Fixed and permeabilized FNA sample of a MCT showed effective separation of mast cells from non-mast cells **(D)**.

The anti-CD117 antibody that was used in this study (ACK45) has been extensively studied in the past and its capacity to label canine mast cells has been reported (19). Nonetheless, cell surface receptor staining protocols (which have been previously reported) (19) do not require cell fixation and permeabilization, which can impact cell staining properties. As the goal is to label CD117 and Ki-67 (intra-nuclear transcription factor) concurrently, fixation and permeabilization steps had to be incorporated into the protocol. The anti-CD117 antibody produced a strong signal when applied to the NI-1 cells without fixation, as expected (Figure 1A). When NI-1 cells were spiked with canine whole blood followed by subsequent fixation and permeabilization, the high CD117 expression of NI-1 cells formed a discrete population that could be readily separated from the peripheral blood leukocytes (Figure 1B). A fixed and permeabilized peripheral blood sample from a dog with mastocytemia (10% mast cells on a 100-cell differential count) showed adequate separation of mast cells from other leukocytes (Figure 1C). This anti-CD117 antibody was further effective in differentiating mast cells from non-mast cells in an FNA sample that was fixed and permeabilized from a dog with MCT (Figure 1D).

### pKIT staining yields a non-specific staining pattern

The polyclonal 5401R anti-pKIT antibody showed significant non-specific staining of NI-1 cells and peripheral blood leukocytes (data not shown). Consequently, pKIT was excluded from the final FC panel.

### The MIB-1 anti-Ki-67 antibody provides a superior staining quality of canine neoplastic mast cells

**Figure 2:**
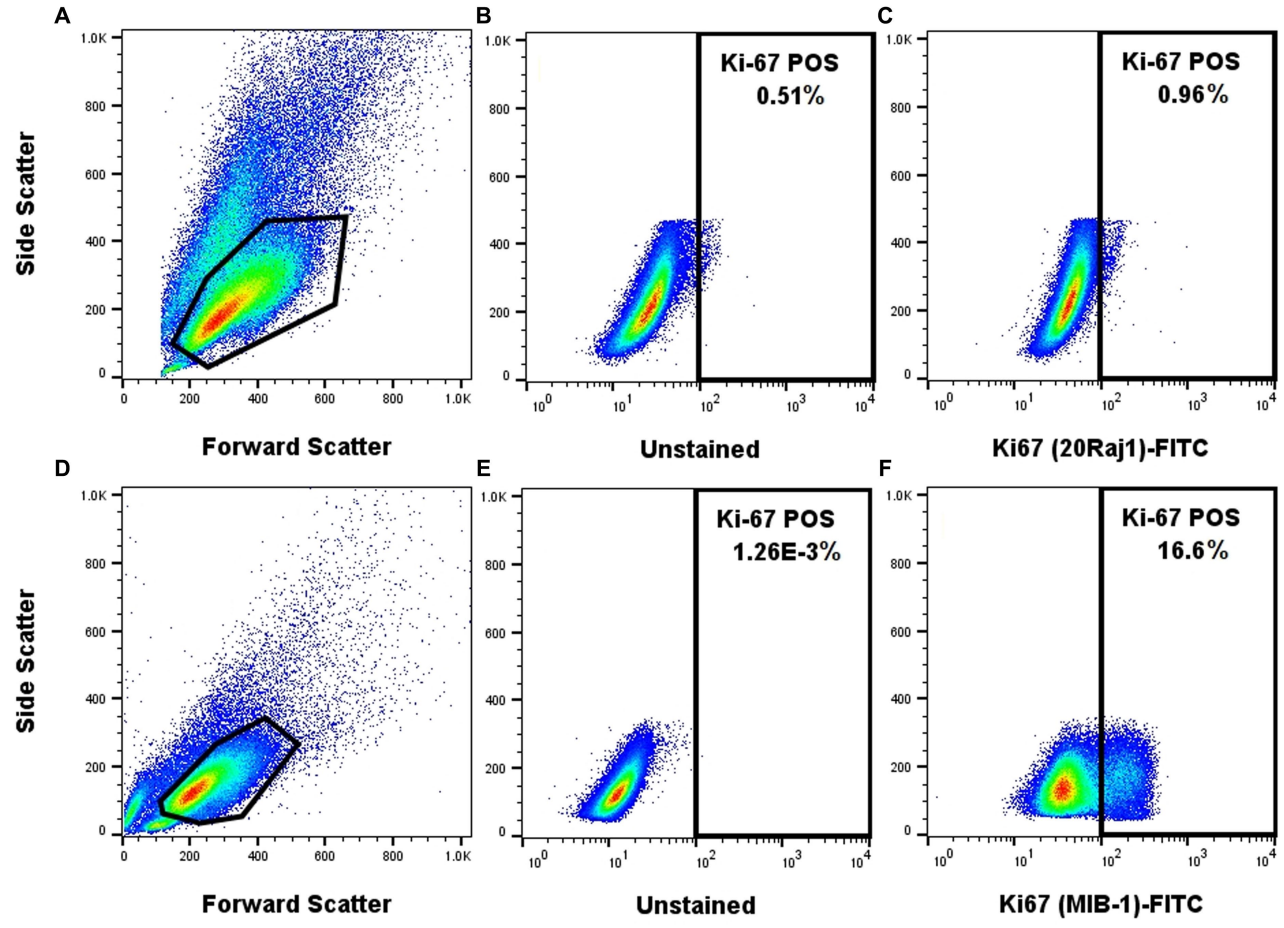
The MIB-1 anti-Ki67 antibody provides a superior staining quality of NI-1 canine neoplastic mast cells. NI-1 cells were either stained with the 20Raj1 clone **(A–C)** or MIB-1 clone **(D–F)**.

The MIB-1 clone of the anti-Ki-67 antibody produced a clearly identifiable and distinct signal in NI-1 cells using the FoxP3 transcription factor fix/perm buffer set (Figure 2). On the other hand, the 20Raj-1 clone of the anti-Ki-67 antibody did not produce credible staining in NI-1 cells, despite the many protocol modifications to the fix/perm reagents, concentration, incubation time, and incubation temperature (Figure 2). It also did not produce credible staining of clinical MCT FNA samples (data not shown). However, it did produce positive staining on peripheral blood lymphocytes and one case of suspected acute leukemia (Supplementary Figure S2).

### Proof of concept application of our FC panel

**Figure 3:**
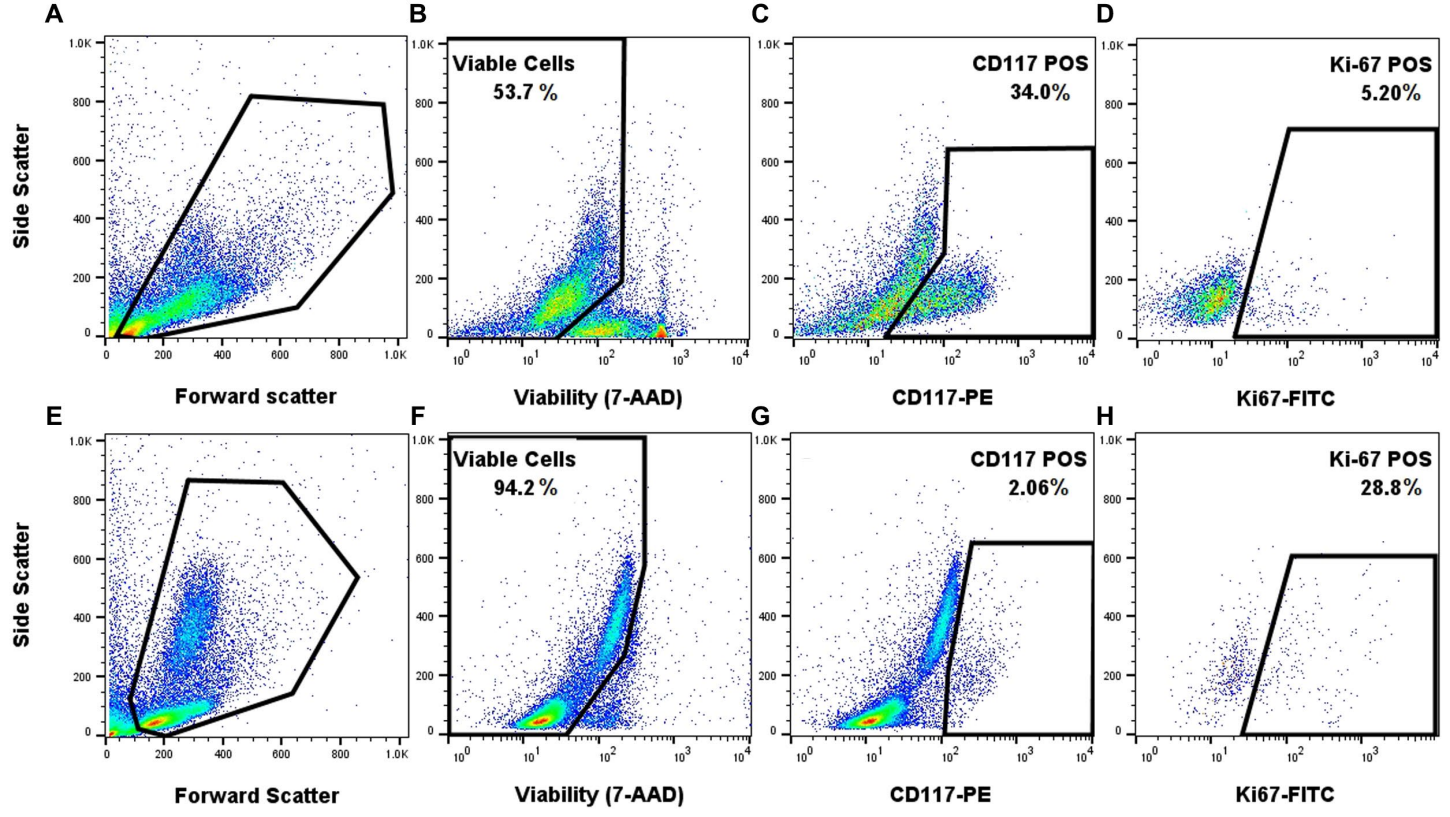
Proof of concept application of the FC panel using samples from a low-grade canine cutaneous MCT **(A–D)** and a biologically aggressive MCT **(E–H)**. Forward and side scatter for the two different samples **(A,E)**. Gating on the viable cells using 7-AAD **(B,F)**. Gating on CD117 positive cells within the viable cells **(C,G)**. Gating on viable and CD117 positive cells that express Ki-67 **(D,H)**. Appropriate single-color and FMO controls were used.

Once the staining protocol for each individual marker was optimized, they were combined to determine if appropriate staining patterns could be identified in FNA samples of clinical MCTs. Appropriate single-color and FMO controls were used. Application of the FC panel to a histologic grade 1/low grade tumor and to a biologically aggressive tumor (confirmed metastasis to the lymph node on cytology) produced a distinct Ki-67 signal that was low (5.2%) in the low-grade tumor sample and was markedly higher (28.8%) in the biologically aggressive tumor (Figure 3). The biologically aggressive tumor had a low number of mast cells on cytology, consistent with the FC results. A 500-cell differential was performed on a Wright Giemsa stained slide consisting mostly of neutrophils with fewer macrophages, lymphocytes, and eosinophils and 1.6% well-granulated mast cells.

## Discussion

Canine cutaneous MCTs have a highly variable biological behavior and the subcutaneous tumors could be aggressive as well. Limited pre-surgical options are available to assess their prognosis and guide further staging and treatment. The current study developed a FC panel that enables the quantification of Ki-67 expression, a validated IHC prognostic marker, in viable neoplastic mast cells using FNA samples that can be applied pre-surgically. Future studies will be required to determine its clinical efficacy for canine MCT prognostication.

Cell yield experiments showed that sufficient cells can be readily obtained from mast cell tumors via FNA, with 78% of the cases having a total cell yield well above the estimated 150,000 cells needed for this panel (Supplementary Figure S1). This reflects the results of a previous report, where 34/38 (89%) FNA samples of MCT yielded sufficient cells (19). Unlike this previous report, the majority (6/9) of our samples were obtained during the pre-surgical visit as opposed to post-surgical excision. Moreover, all of our samples were obtained by the FNA technique and none were supplemented with tumor scraping. Two out of our nine cases (22%) did not yield sufficient cells. Both of these samples came from small tumors (longest axis 1.2 cm and 0.5 cm) and were located on the limbs. One FNA was obtained prior to surgery and the other was obtained immediately post-surgery. Unfortunately, the low number of samples with insufficient cell numbers (*n* = 2) did not allow us to determine if there were any statistical associations. Lastly, different operators collecting the FNA samples may have further contributed to variation in cell yield. Further studies are needed to confirm these observations and offer more guidance on FNA sampling for FC.

To ensure only live cells were assessed for biomarker expression, the DNA-binding dye 7-amino-actinomycin D (7-AAD) was applied. In the current protocol, 7-AAD showed a bright and effective signal that differentiated viable cells from non-viable cells. Although typically used for cell-surface-only staining protocols, 7-AAD previously has been reported for intracellular staining with subsequent cell fixation and permeabilization (27). The alternate intracellular amine-reactive dye (FVS620) produced poor CD117 staining index in clinical mast cell tumor samples and was not included in our final panel (data not shown).

Two different anti-Ki-67 antibody clones (20Raj1 and MIB-1) were tested, including different fixation/permeabilization protocols. The MIB-1 clone showed superior staining of mast cells using the FoxP3 transcription factor fix/perm set (Figure 2). The NI-1 cells (Figure 2) and clinical mast cell tumor samples (data not shown) were not reactive to the 20Raj1 antibody, despite different fixation/permeabilization reagents, incubation time, and incubation temperatures applied. This is in contrast to a study reporting the successful use of the 20Raj1 antibody in flow cytometric analysis of canine lymphocytes (26). Moreover, the immunoreactivity of this antibody was detected in a blood sample from a dog with acute leukemia (Supplementary Figure S2), demonstrating that the antibody can detect the Ki-67 antigen in canine cells. The different results with these two antibodies may be due to specificity to different epitopes or that 20Raj1 antibody may require a different type of cell permeabilization. The cause of this discrepancy was not further investigated as it is outside the scope of this project.

A polyclonal pKIT antibody (5401R, BIOSS) showed broad non-specific and uniform binding to peripheral blood leukocytes (data not shown), which should not express CD117 nor its phosphorylated form. Potential causes for this significant non-specific staining include this being a polyclonal antibody, and also targeting a phosphorylated target, which may require a different type of permeabilization (such as methanol-based) that may not be compatible with the rest of our panel markers. As such, this marker was not included in the final multicolor panel.

Finally, and most importantly, the application of the combined multicolor panel showed that a biologically aggressive mast cell tumor, with cytologic evidence of metastasis to the draining lymph node, had a much higher Ki-67 expression compared to a low-grade tumor (Figure 3). Moreover, this case also demonstrated the robustness of the panel and the biomarkers, where the FC was able to delineate the low percentage of mast cells from the predominating leukocytes. Indeed, the primary tumor was markedly inflamed with only a few mast cells identified on cytologic evaluation (1.6% mast cells in a 500-cell differential count), which is consistent with the low percentage of mast cells on FC (2.06% CD117+ cells). More samples will be needed to determine if there is a significant difference in Ki-67 expression level between low-grade and high-grade tumors and if it can be used for disease prognostication.

There are several limitations to this panel development. First, the low number of FNA samples prevents meaningful assessment if the tumor size and tumor location affects FNA cell yield. Second, multiple operators obtaining the FNA samples can lead to variation in cell yield. Third, the NI-1 cell line is from a dog with mast cell leukemia, not a cutaneous mast cell tumor, and may not have the same pattern of biomarker expression. Lastly, spiked peripheral blood leukocytes were used as a negative control population with the NI-1 cells, but they do not represent the typical leukocyte population (mostly eosinophils) in mast cell tumors.

Nevertheless, we provide proof-of-concept data using clinical FNA samples demonstrating the robustness of our FC panel, its ability to separate neoplastic mast cells from non-mast cells, and effectively label Ki-67 within this neoplastic cell population. The development of a multicolor FC panel on canine cutaneous MCT that includes Ki-67 gives us a powerful tool to assess tumor behavior prior to surgery. The immediate next step would be to examine if there is a correlation between Ki-67 expression in neoplastic mast cells via FC and histologic grade, tumor stage and survival.

## Data availability statement

The raw data supporting the conclusions of this article will be made available by the authors, without undue reservation.

## Ethics statement

The animal studies were approved by Institutional Animal Care and Use Committee University of California, Davis. The studies were conducted in accordance with the local legislation and institutional requirements. Written informed consent was obtained from the owners for the participation of their animals in this study.

## Author contributions

BW: Conceptualization, Data curation, Formal Analysis, Investigation, Methodology, Validation, Visualization, Writing – original draft, Writing – review & editing, Funding acquisition. AL: Conceptualization, Funding acquisition, Methodology, Writing – review & editing. VA: Conceptualization, Funding acquisition, Writing – review & editing. GI: Methodology, Writing – review & editing. FR: Formal Analysis, Methodology, Visualization, Writing – review & editing. AK: Conceptualization, Formal Analysis, Funding acquisition, Investigation, Methodology, Project administration, Resources, Software, Supervision, Validation, Visualization, Writing – review & editing.

## Supplementary material

### SUPPLEMENTARY FIGURE S1

Cell yield from FNA of nine cases of canine MCT. The black dots represent the six cases in which FNA samples were obtained before surgery. The blue dots represent the three cases in which FNA samples were obtained immediately after surgery. The solid black line represents the median total viable cell yield. The dotted black line represents the estimated minimum cell yield required to perform this FC panel.

### SUPPLEMENTARY FIGURE S2

20Raj1 anti-Ki67 antibody labeling of peripheral blood cells of a dog with acute leukemia. Forward and side scatter **(A)**. Unstained control **(B)**. 20Raj1 stained sample **(C)**. Appropriate single-color and FMO controls were used.

### SUPPLEMENTARY TABLE 1

Data collected for all nine cases of canine mast cell tumors.
