# Supplementary figures and images for: Development of a flow cytometric panel to assess prognostic biomarkers in fine needle aspirates of canine cutaneous or subcutaneous mast cell tumors
Source: Front Vet Sci. 2023 Nov 21;10:1279881. doi: 10.3389/fvets.2023.1279881 (PMC10704158; doi:10.3389/fvets.2023.1279881)

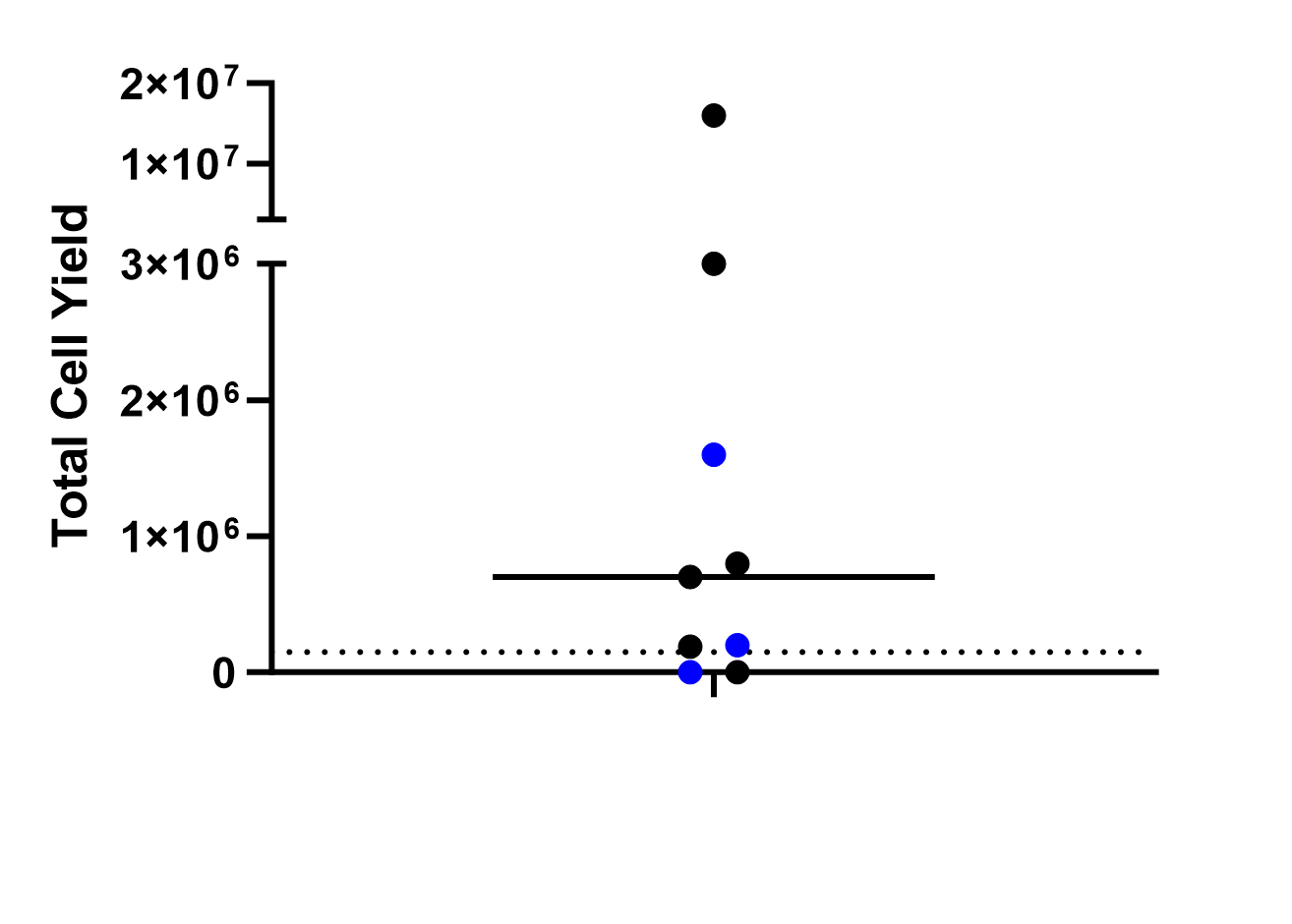

Supplement: Supplementary file 1 [file Image_1.JPEG]

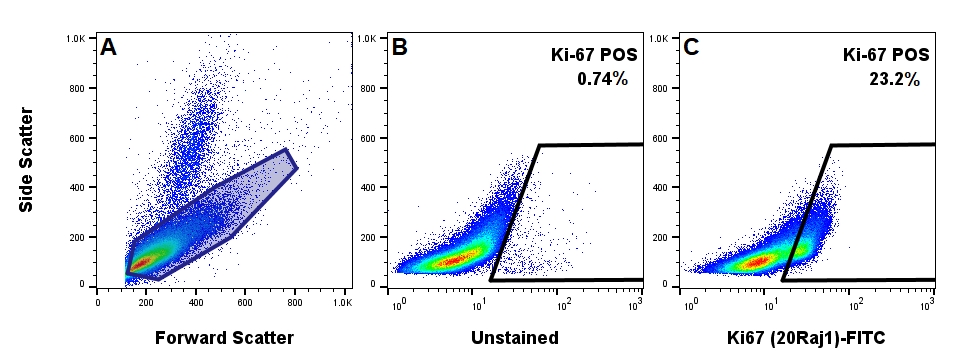

Supplement: Supplementary file 2 [file Image_2.JPEG]
